# Nutritional Keys for Intestinal Barrier Modulation

**DOI:** 10.3389/fimmu.2015.00612

**Published:** 2015-12-07

**Authors:** Stefania De Santis, Elisabetta Cavalcanti, Mauro Mastronardi, Emilio Jirillo, Marcello Chieppa

**Affiliations:** ^1^Laboratory of Experimental Immunopathology, IRCCS “De Bellis”, Castellana Grotte, Italy; ^2^Department of Gastroenterology, IRCCS “De Bellis”, Castellana Grotte, Italy; ^3^Department of Basic Medical Sciences, Neuroscience and Sensory Organs, University of Bari, Bari, Italy; ^4^Istituto Comprensivo Bregante-Volta, Monopoli, Italy

**Keywords:** inflammation, intestinal permeability, nutrition, mucosal immunity, microbiota

## Abstract

The intestinal tract represents the largest interface between the external environment and the human body. Nutrient uptake mostly happens in the intestinal tract, where the epithelial surface is constantly exposed to dietary antigens. Since inflammatory response toward these antigens may be deleterious for the host, a plethora of protective mechanisms take place to avoid or attenuate local damage. For instance, the intestinal barrier is able to elicit a dynamic response that either promotes or impairs luminal antigens adhesion and crossing. Regulation of intestinal barrier is crucial to control intestinal permeability whose increase is associated with chronic inflammatory conditions. The cross talk among bacteria, immune, and dietary factors is able to modulate the mucosal barrier function, as well as the intestinal permeability. Several nutritional products have recently been proposed as regulators of the epithelial barrier, even if their effects are in part contradictory. At the same time, the metabolic function of the microbiota generates new products with different effects based on the dietary content. Besides conventional treatments, novel therapies based on complementary nutrients are now growing. Fecal therapy has been recently used for the clinical treatment of refractory *Clostridium difficile* infection instead of the classical antibiotic therapy. In the present review, we will outline the epithelial response to nutritional components derived from dietary intake and microbial fermentation focusing on the consequent effects on the integrity of the epithelial barrier.

## Introduction

The intestinal tract is the largest interface between the body and the external environment represented by the intestinal lumen (1). The structure of the intestinal wall consists of a mucosa that is highly specialized in each part of the intestinal tract. In the small and large intestine, the mucosa consists of a single monolayer of epithelial cells critical to both absorb nutrients and avoid the entry of potentially harmful entities, including microorganisms or dietary antigens (2). To concomitantly fulfill both functions, the intestinal barrier permeability is regulated by a dynamic process. The entry of small amounts of nutritional antigens and microorganisms can occur even without a pathogenic response. This event induces a homeostatic immune response characterized by immune tolerance to these antigens (3). Intestinal barrier damage can be primarily due to an enhancement of the paracellular permeability associated with an increased permeation of luminal antigens. These events in turn cause the activation of the mucosal immunity finally leading to sustained inflammation and tissue damage (4). Intestinal permeability is a dynamic process whose regulation is determined by the interaction among several players, including barrier constituents, immune cells, microbiota, and also external factors, such as the diet. Alterations of mucosal barrier function are increasingly linked to a broad spectrum of pathologies (5–7), including intestinal disorders such as inflammatory bowel disease (IBD) (8). For this reason, a deeper understanding of the multiple mechanisms involved in the regulation of the mucosal barrier is needed. The main focus of this review is to summarize how nutrition can influence the intestinal barrier function.

## The Intestinal Barrier

**Figure 1:**
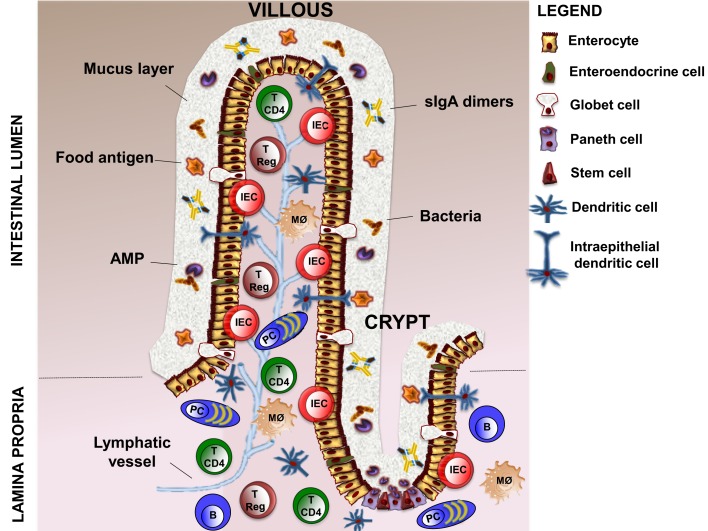
**Structure of intestinal barrier**. The intestinal barrier is a complex entity, which is composed of cellular and extracellular elements. The cellular part is defined by intestinal epithelium (five distinct type of cells, such as stem cells, Paneth cells, enterocytes, goblet cells, and enteroendocrine cells) and the underlying lamina propria, which contains DCs (also intraepithelial DCs, IEDCs), macrophages, intraepithelial lymphocytes (IEL), T regulatory cells (T Regs), TCD4^+^ lymphocytes (T CD4), B lymphocytes (B), and plasma cells (PCs). The extracellular component consists in a mucus layer produced by Globet cells, AMPs secreted by Paneth cells, and sIgA dimers released by plasma cells. DCs, dendritic cells; AMPs, antimicrobial peptides; sIgA, secretory ImmunoglobulinA.

The intestinal barrier is an heterogeneous entity that is composed of cellular and extracellular components (9). The cellular part is defined by the intestinal epithelium and the underlying lamina propria. The intestinal epithelium contains five distinct types of cells: stem cells with interposed Paneth cells producing antimicrobial peptides (AMPs), absorptive enterocytes, mucus secreting goblet cells, and hormone producing enteroendocrine cells (10). Lamina propria contains dendritic cells (DCs), intraepithelial DCs (IEDCs), macrophages, intraepithelial lymphocytes (IEL), T regulatory cells (T Regs), TCD4^+^ lymphocytes, B lymphocytes, and plasma cells (11, 12). The extracellular component of the barrier is represented by the mucus layer secreted by goblet cells. The mucus is crucial to facilitate food passage, to protect the epithelial cells from the action of digestive enzymes present in the lumen, and to avoid the firm adhesion of bacteria to the epithelial cells, thus preventing their entry into the lamina propria (13). This function is implemented by AMPs and secretory (s) IgA dimers released by plasma cells (14) (Figure 1).

The intestinal barrier integrity is essential for the intestinal permeability; specifically, intestinal epithelial cells (IECs) have a major role in this context (15, 16). The epithelial monolayer represents a physical barrier due to the fact that in the absence of specific transporters the plasma membrane of these cells is impermeable to the majority of hydrophilic solutes (9). Epithelial cells are held together by the apical junctional complexes, consisting of adherent junctions and tight junctions (TJs), as well as by underlying desmosomes (17). Adherent junctions and desmosomes are a site of intercellular communication that close together adjacent IECs providing strong bonds between them without altering paracellular permeability as in the case of TJs. TJs are composed of four transmembrane (TM) proteins: such as claudins (18), occludin (19), junctional adhesion molecules (JAMs) (20), and tricellulin (21), and some cytosolic scaffold proteins, such as zonulae occludens (ZO) and cingulin (22, 23). The extracellular domains of the TM proteins form a selective barrier through both homophilic and heterophilic interactions with nearby cells. Conversely, the intracellular domains of TM proteins are linked to the perijunctional actomyosin ring by the interaction with cytosolic scaffold proteins (24). This interaction permits the cytoskeletal regulation of TJ-mediated barrier integrity (17). TJs are also important in determining the charge selectivity of the paracellular pathway (25). Specifically, this effect is exerts by claudins (26). In fact, in the intestine, claudin-2 forms cation-selective channels in TJs (27). TJs are highly regulated at transcriptional and post-transcriptional levels by physiological or pathophysiological stimuli (9); among these, an important role has been attributed to nutrients, as discussed below.

## Intestinal Permeability and Transport Pathways

Intestinal permeability is an intrinsic property of the intestine that is defined as “the facility with which intestinal epithelium allows molecules to pass through by non-mediated passive diffusion” (28). The transport of molecules from the intestinal lumen to the lamina propria can occur through two different pathways: the paracellular diffusion of small molecules by TJs and the transcellular transport through transcytosis (endocytosis/exocytosis) of large molecules mediated or not by membrane receptors (29). Both pathways allow luminal antigens to gain access to the subepithelial compartment and to interact with local immune cells.

Paracellular permeability is mainly determined by pore size in TJs, determining a high-capacity pathway that is size-restricted and a low-capacity pathway, independent of size. The latter can be due to transient (e.g., apoptosis) or fixed breaks (different TJ proteins) in the epithelial cells (29). In fact, small and large pores are defined by different TJ proteins, such as claudins (30, 31) and tricellulin (17), respectively. The paracellular diffusion of small molecules through TJ pores is driven by water movement in response to electrochemical or osmotic gradients across the epithelium (29).

Transcellular transport pathways can be mediated by different type of cells; M cells overlying Payer’s patches (PPs) and isolated lymphoid follicles (32), DCs (33–35), goblet cells (36), and columnar enterocytes. Enterocytes sample molecules of high molecular weight, such as food antigens, by endocytosis at the apical membrane and transcytosis toward the lamina propria (37, 38). During transcytosis, full-length proteins are degraded in acidic and lysosomal compartments of enterocytes and released at the basolateral membrane as amino acids when totally degraded or as immunogenic peptides if partially degraded (39). Immunogenic peptides released into the lamina propria can be taken up by local antigen-presenting cells (APCs) and activate the immune response (40); alternatively, they can be transported into the draining lymph nodes by lacteals (41). The presence of food antigens in the form of small immunogenic peptides suggests that during transcytosis, a mechanism that avoids their total degradation might occur (42). This could be due to exosome-like structures produced by IECs. Exosomes are small membrane vesicles (~80 nm in diameter) resulting from inward membrane invagination of the MHC class II-enriched compartments in which luminal antigens arrived after endosomal degradation (43). These compartments can both lead to lysosomes or fuse with the plasma membrane. Exosomes released outside the cells interact with local immune cells (44). Exosome-bound peptides are more efficient in interacting with DCs and promoting antigen presentation to T cells than free peptides (45). Also, in humans, it was demonstrated that HLA-DR/peptide complexes bound to exosome-like vesicles were highly immunogenic (46).

The transcytosis of food antigens occurs primarily by a fluid-phase endocytosis of proteins at the apical membrane of enterocytes (29). However, the access of luminal antigens into the intestinal mucosa is also possible thanks to the expression of immunoglobulin receptors (IgR) expressed on the apical surface of enterocytes (47). After the binding to IgRs, luminal antigens cross the barrier in the form of immune complexes (ICs) (29). IgA is the most representative Ig isotype at the mucosal interface, and it is secreted in the intestinal lumen through polymeric IgR (pIgR) in the dimeric form of sIgA (48). sIgA represents one of the mechanisms exerted by intestinal barrier to control the interaction of microbial and food antigens with the intestinal lumen (29). Despite the usual basal-to-apical secretion pathway of the sIgA (48), in some pathological conditions, an abnormal retro-transport of sIgA ICs can allow the entry of luminal antigens in the intestinal mucosa (29). The IgA-mediated retro-transport of pathogenic bacteria is beneficial for bacterial clearance and intestinal homeostasis (49). The same mechanism applied to non-pathogenic antigens, such as food antigens, could be deleterious rather than protective (48). One of the most common examples of the aforementioned mechanism is celiac disease (CD), an enteropathy induced by the abnormal activation of T cells by gluten-derived gliadin peptides (50). The high proline content of gliadin prevents their efficient digestion and leads to the release of large immunogenic peptides that cause CD (51). The ectopic expression of the transferrin receptor CD71 at the apical surface of IECs of CD patients allows the retro-transport of sIgA/gliadin IC into the lamina propria (29). On the contrary, in healthy individuals, CD71 is expressed on basolateral membrane of IECs and this permits gliadin peptides to be almost totally degraded (52). Also, IgEs are involved in transcytosis of food antigens due to the expression of their receptor, CD23, at the apical side of enterocytes (53). Increased expression of this receptor can drive the transport of IgEs/allergen ICs from the intestinal lumen to the lamina propria causing mast cell degranulation and allergic inflammatory reactions (54).

## Regulation of the Intestinal Barrier Function by Nutritional Means

Food is not only a source of nutrients but may also modulate some physiological functions of the body. This is especially true for the intestinal tract because of the continuous interaction of the intestine with dietary antigens (55). Recent studies demonstrated the effects of the interaction between food and IECs. In fact, dietary antigens are able to modulate transporter activity, TJ permeability, metabolic enzyme expression, immune functions, and microbiota (56).

### Food Regulation of Transporters and Ion Channel Function

Absorption of nutrients is mainly located in the small intestine, while maintenance of fluid-ion homeostasis mostly happens in the large intestine. This process is regulated by transporters and ion channels localized on the enterocytes membrane.

Glucose absorption in the small intestine is strictly regulated by glucose transporters in IECs that are distinguished as sodium dependent (SGLT1) and sodium independent (GLUT2, 3, 5) (57). The inhibition of these transporters is induced by some food derivates, such as polyphenolic compounds [tannic acid, chlorogenic acid, catechins, in particular, epigallocatechin gallate (EGCG)] (58, 59) and gymnemic acid, a taste-modulating triterpene glucoside extracted from the leaves of *Gymnema sylvestre* (60). Also, calcium uptake by specific transporters located on the IECs is regulated by diet compounds (61). For example, enhanced calcium uptake is observed with whey protein digest though the mechanism is not defined at all (62).

Furthermore, most of the ion channels present in epithelial cells are regulated by the levels of cyclic nucleotides, especially cAMP. Food indirectly regulates the levels of cAMP by augmenting the secretion of some hormones (63, 64), such as guanylin and natriuretic peptides, that increase cAMP levels. cAMP can directly bind to cyclic nucleotide-gated ion channels (CNG channels) and induce the influx of calcium ions into the cells (65). It can also inhibit the activity of sodium–hydrogen exchanger3 (NHE3), thus preventing the absorption of sodium ions, chloride ions, and water by the epithelium (66–68).

### Food Regulation of TJ Permeability

Tight junctions are not static but highly dynamic structures constantly shaped due to interactions with internal/external stimuli, such as cytokines, growth factors, food residues, and pathogenic and commensal bacteria (56).

**Figure 2:**
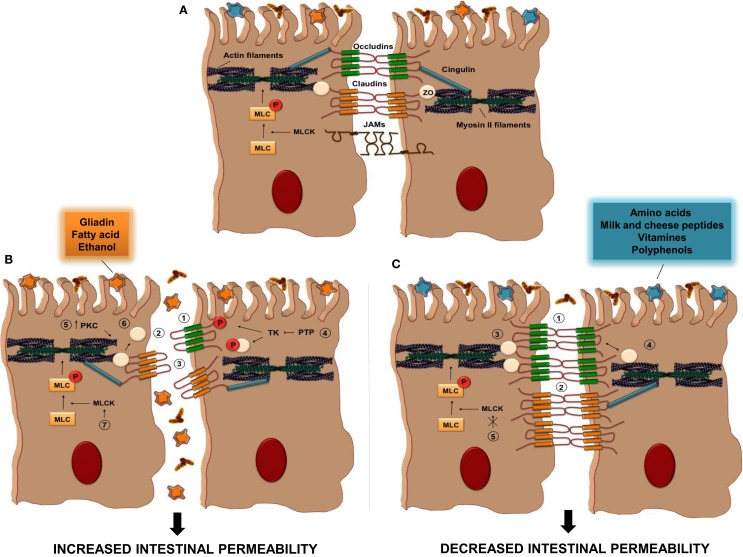
**Tight Junction regulation by food antigens**. **(A)** TJs are composed of some transmembrane proteins [occludin, claudins, and junctional adhesion molecules (JAMs)] and cytosolic scaffold proteins [zonulae occludens (ZO) and cingulin]. The extracellular domains of transmembrane proteins of adjacent IECs interact to form the selective intestinal barrier, while cytosolic scaffolds anchor the transmembrane proteins to the actomyosin ring. **(B)** The intake of some food antigens, such as gliadin, fatty acids, or ethanol, can directly increase intestinal permeability by different mechanisms (1–7); (1) alteration in cellular distribution of occludin proteins, (2) reduction in the cellular content of occludins, (3) alteration in cellular distribution of claudin, (4) inhibition of protein tyrosine phosphatase (PTP) activity that induce tyrosine phosphorylation of ZO-1 and occludin and their dissociation from the junctional complex, (5) activation of PKC that leads to polymerization of actin and subsequent displacement of TJ proteins, including ZO-1, (6) displacement of ZO proteins from the junctional complexes, (7) activation of MLCK activity. **(C)** Other dietary antigens, such as amino acid, milk and cheese peptides, vitamins, and polyphenols, have the ability to decrease intestinal permeability through distinct pathways; (1–3) increase in the cellular content of occludin, claudin, and ZO proteins, respectively, (4) restoration of ZO-1/occludin assembly, (5) inhibition of MLCK activation. PKC, protein kinase C; MLC, myosin light-chain; MLCK, myosin light-chain kinase; TK, tyrosine kinase.

Food substances can act by increasing or decreasing TJ permeability, as described in Figure 2 and summarized in Table 1. One of the most common parameters to evaluate the intestinal permeability, as will be described later, is transepithelial electrical resistance (TER) that measure paracellular ions flux (69). Among the food-derived compounds able to modulate the intestinal barrier function, there are some amino acids. Glutamine (Gln) represents the primary source of amino acids for the intestinal mucosa (17). It was demonstrated that Gln improves intestinal barrier function in highly stressed patients (70) and in animal models of endotoxin-induced permeability (71). Also, Gln can restore stress-induced loss of barrier integrity by increasing TER (72). The increase in permeability was confirmed in another work in which Caco-2 cells were deprived of Gln by a combination of a Gln-free media and the inhibition of Gln synthetase (73).

**Table 1:** **Effect of diet-derived compounds on intestinal permeability**.

Dietary antigens	Effect on permeability	TER measurement	Mechanisms of action	Models of study	Reference
**AMINO ACIDS**					
Gln	Decreased	Increased	Unknown	Caco2 cell line	(66)
Gln deprivation	Increased	Not determined	Reduction of occludin, claudin-1, and ZO-1/redistribution of claudin-1 and occludin	Caco2 cell line	(67)
Trp	Decreased	Increased	Unknown	Caco2 cell line	(70)
**PEPTIDES**					
β-casein	Decreased	Increased	Increase occludin expression	Caco2 cell line	(71)
β-lactoglobulin	Decreased	Increased	Modifications into the cytoskeletal structure	Caco2 cell line	(72)
**VITAMINS**					
Vitamin D	Decreased	Increased (in Caco2 cell line)	Enhancement of claudin-1, ZO-1 and E-cadherin proteins expression	SW480-Caco2 cell lines/VDR^+/+^ and VDR^−/−^ in C57BL6 background	(73)
Retinol (vitamin A)	Decreased	Increased	Neutralization *Clostridium difficile* toxin A	Caco2 cell line	(74)
**POLYPHENOLS**					
Quercetin	Decreased	Increased	Increase in claudin-4 expression and in ZO-2, occludin and claudin-1 assembly	Caco2 cell line	(75, 76)
Kaempferol	Decreased	Increased	Promotion of ZO-1/2, occludin and claudin-1/3/4 cytoskeletal association	Caco2 cell line	(77)
Genistein	Decreased	Increased	Inhibition of the redistribution and the dissociation of occludin/ZO-1 complex	Caco2 cell line	(78, 79)
	Decreased	Increased	Inhibition of TNFα-mediated effects	HT-29/B6 cell line	(99)
EGCG	Decreased	Increased	Inhibition of INFγ-mediated effects	T84 cell line	(98)
Curcumin	Decreased	Increased	Inhibition of TNFα- and IL-1β-mediated effects	Caco2 cell line	(100, 101)
**DITERPENE GLYCOSIDE**					
Capsianoside	Increased	Decreased	Changes in F/G actin ratio	Caco2 cell line	(92)
**LCFAs**					
EPA and DHA	Increased	Decreased	Protein kinase C regulation/unknown	Caco2 cell line	(84, 85)
	Decreased	Increased	Reduction of IL-4-mediated permeability	T84 cell line	(86)
**MCFA**					
Capric acid	Increased	Decreased	Redistribution of occludin and ZO-1/MLCK activation	Caco2 cell line	(87)
Lauric acid	Increased	Decreased	MLCK activation	Caco2 cell line	(88)
**SCFAs**					
Acetic and propionic acids	Decreased	Increased	Activation of PI3K	Caco2/T84 cell lines	(90)
**MINERALS**					
Zinc depletion	Increased	Decreased	Redistribution of occludin, ZO-1, E-cadherin, and β-catenin and F-actin	Caco2 cell line	(93)
**ALCOHOLS**					
Ethanol	Increased	Decreased	Redistribution of occludin and ZO1/MLCK activation	Caco2 cell line	(94)
Acetaldehyde	Increased	Decreased	Loss of interaction between occludin/ZO-1 and β-catenin/E-cadherin by a tyrosine phosphorylation-dependent mechanism	Caco2 cell line/Sprague-Dawley rats and C3H/He mice	(79, 95–97)
Chitosan	Increased	Decreased	Redistribution of ZO-1 and F-actin distribution	Caco2 cell line	(91)

The barrier impairment is caused by the perturbation of TJ protein (ZO-1, occludin, and claudin-1) expression and distribution. These mechanisms are mediated by the PI3K/Akt pathway since the genetic knockdown or pharmacological inhibition of PI3K is able to neutralize TER reduction induced by Gln deprivation (74). Furthermore, Gln can also prevent the negative effects of acetaldehyde, an oxidized metabolite of ethanol, avoiding the redistribution of ZO-1 and occludin. This action requires the activation of EGF receptor (75). Another amino acid, tryptophan (Trp), reduces intestinal permeability by increasing TER in Caco-2 cells in a dose-dependent manner even if the molecular mechanism is not yet known (76). Furthermore, peptides derived from cheese and milk proteins have been shown to suppress intestinal permeability. β-casein peptide increases both occludin expression and TER in Caco-2 (77), while β-lactoglobulin induces an increase in TER likely due to changes into the cytoskeletal structure. In fact, treatment of cells with cytochalasin D (able to disrupt the cytoskeleton) inhibits TER increase induced by β-lactoglobulin (78).

Also, some vitamins show protective effects on intestinal permeability. For example, vitamin D enhances the expression of TJ (ZO-1 and claudin-1) and adherent junction (E-cadherin) proteins (79). Vitamin D is also able to prevent dextran sodium sulfate (DSS)-induced decrease in TER both *in vitro* and *in vivo* models in a vitamin D receptor (VDR)-dependent manner (79). In fact, VDR knockout mice exhibits more severe colitis as compared to wild-type mice due to earlier intestinal barrier defects than wild-type mice, as indicated by TER and TJ proteins expression. Retinol, an alcohol form of vitamin A, partially attenuates the decreases in TER induced by *Clostridium difficile* toxin A in intestinal Caco-2 cells (80). However, the underlying mechanism remains to be elucidated.

Polyphenols participate in the regulation of the intestinal barrier too. The flavonoid subgroup, quercetin, myricetin, and kaempferol, enhances barrier integrity in intestinal Caco-2 cells (17). Quercetin, the most common flavonoid in nature, increases TER and reduces paracellular flux across Caco-2 monolayers in a dose-dependent manner (81, 82). This mechanism is accompanied by an increase in claudin-4 expression and the assembly of ZO-2, occludin, and claudin-1 at the TJ level. Also, kaempferol increases TER due to promotion of the cytoskeletal association of ZO-1, ZO-2, occludin, claudin-1, claudin-3, and claudin-4 and an increase in the expression of some TJ proteins (83). The isoflavonoid genistein inhibits the redistribution and the dissociation of occludin/ZO-1 complex protecting barrier integrity against acetaldehyde and oxidative stress (84, 85).

On the contrary, other food compounds negatively regulate intestinal barrier, e.g., gliadin whose effect correlates with CD (86), as previously discussed. Gliadin binds to CXCR3 on IECs and increases intestinal permeability by a MyD88-dependent release of zonulin (87). Zonulin interacts with a specific surface receptor activating phospholipase C that hydrolyzes phosphatidyl inositol releasing inositol 1,4,5-trisphosphate (IP3) and diacylglycerol (DAG) (88). DAG and PPI-3 can directly and indirectly (through the release of intracellular Ca^2+^), respectively, activate protein kinase C (PKC). Activated PKC catalyzes the phosphorylation of target proteins with subsequent polymerization of soluble G-actin in F-actin. This polymerization causes the rearrangement of the filaments of actin and the subsequent displacement of proteins, including ZO-1 from the junctional complex. As a result, intestinal TJs become looser allowing the paracellular passage of gliadin from the intestinal lumen to the lamina propria (89). *Ex vivo* experiments on C57BL6 and CXCR3^−/−^ mice confirmed these observations. Intestinal segments exposed to gliadin from wild-type but not from CXCR3^−/−^ mice increase zonulin release and intestinal permeability (87). Furthermore, it has recently been demonstrated by *in vivo* intravital microscopy that gliadin modulates intestinal permeability inducing a redistribution of ZO-1 (90).

Middle-chain fatty acids (MCFAs), e.g., capric acid and lauric acid and long-chain fatty acids [LCFAs, e.g., eicosapentaenoic acid (EPA), γ-linoleic acid, and docosahexaenoic acid (DHA)] are also able to increase TJ permeability by reducing TER (91, 92). The barrier properties of the latter are controversial since it was discovered that, unlike the finding in Caco2 cell monolayer, EPA and DHA were particularly effective in supporting barrier integrity. In a different cell line (T84 cell), administration of EPA and DHA improves resistance and reduces IL-4-mediated permeability (93). Capric acid, but not lauric acid, induced the redistribution of TJ proteins (occludin and ZO-1) and the rearrangement of the cytoskeleton actin (94). This means that these two MCFAs act with a different mechanism (95). The common ground is the paracellular permeability induced by both acids that requires intracellular Ca^2+^-dependent myosin light chain kinase (MLCK) activation (95). Short-chain fatty acids (SCFAs) also modulate intestinal barrier. The SCFAs, such as acetate, propionate, and butyrate, are the major anions in the colon and are mainly produced by bacterial fermentation of undigested carbohydrates. Butyrate strengthens the barrier through the increase among TER, ZO-1/ZO-2, and cingulin protein in rat-1 fibroblasts (96). Acetic and propionic acids from diet increased TER and decreased permeability to lucifer yellow in a dose-dependent manner in the rat colon and intestinal cells (97). The acetic acid-mediated increase in TER could be suppressed by the pharmacological inhibition of PI3K (97).

Apart from fatty acids, there are other food compounds that negatively regulate barrier function. Chitosan, a polysaccharide widely used in the food industry, is able to increase paracellular permeability by altering the distribution of ZO-1 and F-actin (98). Moreover, sweet pepper extract decreases TER, and this effect is likely mediated by capsianoside, its active compound, that induces dysfunctional TJs by changing the F-actin and G-actin ratio (99).

Zinc is essential for the survival and function of the cells and its depletion increases intestinal permeability by reducing TER and altering the expression of ZO-1, occludin, and F-actin filaments (100). Alcohol and its oxidized metabolite, acetaldehyde, impair intestinal barrier function. Ethanol decreases TER and increases mannitol flux due to a redistribution of ZO-1 and occludin (101) and barrier impairment seems to be caused by a MLCK-dependent mechanism. Administration of acetaldehyde *in vivo* induces an impairment of barrier integrity, as indicated by TER decrease and improved dextran permeability (102). *In vitro* studies show that the underling mechanism acts via a tyrosine kinase-dependent mechanism (85, 103). The suppression of protein tyrosine phosphatase (PTP) activity by acetaldehyde causes tyrosine phosphorylation of ZO-1, occludin, E-cadherin, and β-catenin and their dissociation from the respective TJ and AJ complexes, finally leading to increased paracellular permeability (85, 104).

Food components can regulate TJ permeability not only directly by targeting signal transduction pathways involved in TJ regulation but also indirectly by influencing cytokine signaling involved in this modulation (17). For example, epigallocatechin-3-gallate (EGCG), the predominant polyphenol in green tea, does not affect epithelial permeability when administered alone to confluent T84 cells (105). On the contrary, the administration of this polyphenol in combination with IFNγ prevents the negative effects of this cytokine on epithelial permeability (105). Furthermore, genistein inhibits the tumor necrosis factor α (TNFα)-mediated TER reduction in HT-29/B6 cells (106). Finally, it was demonstrated that another polyphenol, curcumin, is able to block TNFα- and inteleukin-1β (IL-1β)-induced NF-κB activation, increasing TER and reducing intestinal permeability (107, 108).

### Food Regulation of Intestinal Detoxification Systems

Detoxification systems in IECs are activated after the binding of xenobiotic to intracellular receptors [e.g., the arylhydrocarbon receptor (AhR) and the pregnant X receptor (PXR)] and the subsequent entry into the nucleus that induce the expression of some detoxification enzymes (109). These enzymes are involved in the oxidation and conjugation of xenobiotics. They are also involved in the excretion of harmful compounds from the cells (110). It was demonstrated that some flavonoids and terpenoids are able to induce PXR-dependent transcriptional activity activating the intestinal detoxification system (111). Moreover, dietary flavonoids can help to balance the ratio between activation and suppression of detoxification enzymes to avoid the possibility that a helpful mechanism could become detrimental by reducing the bioavailability of drugs and functional foods (56).

### Food Regulation by Microbiota

The microbiota is involved in several functions crucial for the host homeostasis. These include metabolic homeostasis and cross talk with the immune system. The metabolic function is fundamental, as the bacterial degradation of some complex nutrients from food is the source of essential amino acids and vitamins (112). In fact, human enzymes cannot degrade the most complex carbohydrates and plant polysaccharides (113). Among the metabolites produced during the process, there are some essential products absorbed by the host. These include vitamins, such as vitamin K, and most of the water-soluble B vitamins, such as biotin, cobalamin, and riboflavin (114, 115). Furthermore, other metabolites produced by microbiota are the SCFAs (as previous discussed), fermentable carbohydrates, and resistant starches, which are not broken down in the upper digestive tract (116). Fermentation of these substrates also results in the production of gases, including hydrogen, methane, and hydrogen sulfide.

In addition, the intestinal microbiota is involved in the activation/inactivation of bioactive food compounds, such as isoflavanoid and plant lignans (112). In fact, a metabolic transformation, including deglycation and hydrolysis, is required for many plant polyphenols to induce their biologically active form. Within the colon, polyphenols are broken down by the microbiota to a variety of small phenolic components whose physiological relevance is not yet fully known (117). In addition, recent studies demonstrated that microbiota composition could be selectively modulated by polyphenol (118). Therefore, the positive effects associated with polyphenols consumption (105, 119, 120) should not only be attributed to their bioactive metabolites but also to the modulation of the intestinal microbiota.

Microbiota is also able to convert prodrugs into their bioactive forms and to modify xenobiotics and bile acids with potential effects on the GI motility, secretion, and immune function (112). These effects are also due to bile acids contribution to the modulation of microbiota composition (121). Among harmful products of protein fermentation, hydrogen sulfide might be relevant for compromising intestinal health. In fact, it can be converted to thiosulfate and further oxidized to tetrathionate during inflammation. This product supports the growth of tetrathionate utilizing pathogens, many of which have been associated with the intestinal symptoms of irritable bowel syndrome (IBS) patients (122).

The metabolic function of the microbiota can result in opposite effects based on the dietary content. Diets containing fermentable fibers increase SCFA-producing bacteria. The produced SCFAs in turn protect the epithelium by acting on the intestinal barrier. Specifically, these diets increase TJ protein production and TER and decrease permeability and bacterial translocation. SCFAs also stimulate IEC metabolism, turnover, and apoptosis (123). A similar effect can be described for diets enriched in probiotic bacteria. On the contrary, diets that favor growth of pathogenic or opportunistic bacteria (as with intake of milk fat) would have opposite effects, compromising barrier integrity by altering TJ proteins production and distribution, and decreasing TER. These effects finally result in increased barrier permeability boosting bacterial translocation (124).

## *In Vitro* and *In Vivo* Models to Study Intestinal Permeability

Gold standards to measure intestinal permeability are the measurement of TER for *in vitro* study and the intestinal permeability test (IPT) for *in vivo* study (29). TER measurement is an easy-to-perform technique to investigate both the functional expression and the regulation of TJs (69). To perform measurements, cells are cultured as monolayers on commercially available permeable filter supports. To better mimic the physiological epithelial cell layer filter, supports have been designed with different pore densities and pore diameters (125). The most frequently used cellular line to study intestinal permeability is Caco-2. This line derives from human epithelial colorectal adenocarcinoma cells (73, 81, 107) and when cultured in appropriate conditions is able to create a monolayer of cells with a luminal and basolateral polarization. Apart from the measurement of TER, *in vitro* studies also included the evaluation of the effects of human biopsy extracts on permeability to fluorescein isothiocyanate (FITC)–dextran in confluent monolayers of Caco-2 cells. Furthermore, to assess high capacity size and charge selective pathway versus low capacity paracellular route the morphological measurements of TJ components in mucosal biopsies and the polyethylene glycol (PEG) profiling were used (126). Moreover, the Ussing chamber technique provides a short-term intestine fragments culture that measure electrical and transport parameters of an intact polarized intestinal epithelium (127). Some concerns with this method are related to the limited viability and function of an *ex vivo* intestinal preparation and the number of measurements that seem to be under representative for the physiological complexity of the intestinal mucosa (128).

For *in vivo* models of intestinal permeability, IPT directly measures the ability of two non-metabolized sugar molecules (lactulose and mannitol) to permeate the intestinal mucosa (129, 130). The degree of intestinal permeability is expressed by the levels of these two sugars recovered in a urine sample collected over the next 6 h (127). In fact, in case of barrier function loss these molecules cross the intestinal barrier, enter into the circulation and can be detected in urine after renal excretion. IPT can discriminate between paracellular (lactulose) and transcellular (mannitol) pathways *in vivo*, and it is a useful test in clinical studies because it gives information on the overall status of the intestine (villous atrophy and inflammation) (29). For research purpose, to study intestinal permeability *in vivo*, experimental animal models can be used. It is possible to test the presence of macromolecular tracers (dextrans or Evans Blue) in the blood after gavage (29). But with this procedure, several parameters that significantly affect the measurement of intestinal permeability, such as gastrointestinal (GI) motility affecting the time of contact of the tracer with the mucosa and body distribution of the tracers, are not be taken into account. For this reason, to measure intestinal permeability in animal models, intestinal loop systems and tracer recovery in mesenteric or portal blood could be preferred even if they require longer and more invasive procedures (131).

## Food Regulation of Intestinal Inflammation: Cooperation Between Diet, Immune System, and Microbiota

Together with the IECs, there are at least two other important components to be taken into account for the regulation of intestinal permeability: one internal and one external. The intestinal immune system accounts for the internal side of the barrier, while the microbiota represents the external one (132). Intestinal immunity is characterized by numerous dynamic responses that contribute to maintain the delicate balance between the capacity of mounting protective immune responses against infectious agents and the ability to tolerate innocuous antigens present in the intestinal lumen (133). The intestinal barrier is not completely impermeable to macromolecules. In fact, in the steady state, the transepithelial passage of small amounts of food-derived antigens and microorganisms participates in the induction of the homeostatic/tolerogenic immune response toward food antigens and commensal bacteria (12, 134). The mucosal immune system of the GI tract is composed of distinct immune cell types, such as neutrophils, monocyte/macrophages, DCs, mast cells, innate lymphoid cells, B, and T cells (135). The intestinal immune system regulates mucosal barrier function by different mechanisms: the modulation of epithelial dynamics, the regulation of AMPs production, the influence on the microbiota, and the induction of a response against microorganisms and luminal antigens that cross the barrier (13). Cytokines secreted by immune cells have been described as mediators involved in the regulation of mucosal barrier function at various levels, including the epithelial integrity and the immune response (136, 137). For example, TNFα, among the major players involved in the inflammatory process, augments paracellular permeability by removing claudin 1 from TJs, increasing claudin 2 expression, and enhancing occludin degradation (138). The inflammatory response can also be modulated by some amino acids. Taurine, one of the most abundant free amino acid in mammals, exerts anti-inflammatory functions both *in vivo* and *in vitro* (139, 140). The effects are mediated by taurine transporters (TAUT) whose synthesis and activity are increased in response to external stresses [e.g., osmotic pressure (141) and inflammatory cytokines (142, 143)] due to the necessity to maintain high intracellular level of this amino acid. Zhao et al. demonstrated that taurine supplementation reduces weight loss, diarrhea severity, colon shortening, and induces an increase in colonic tissue myeloperoxidase activity after DSS treatment (140). They also proved that taurine was able to inhibit the secretion of macrophage inflammation protein-2 (MIP-2) from IECs and the infiltration of inflammatory cells, such as neutrophils (140). Furthermore, several plant-derived polyphenols have been described as modulators of the inflammatory response (82, 119, 120, 144). Their effect is likely mediated by the selective suppression of the inflammatory response of intestinal DCs. Possibly, the DCs mostly affected by the polyphenol exposure are those projecting dendrites into the intestinal lumen (33–35) where these compounds are more abundant.

The microbiota greatly influences mucosal barrier function both by direct and indirect modulation of the epithelial layer and mucosal components (145, 146). Specifically, a role in the regulation of intestinal permeability must be considered for some important microbiota components, i.e., probiotics and prebiotics. Probiotics are defined by the World Health Organization as “live organisms which, when administered in adequate amounts, confer a health benefit on the host” (147). Probiotics are non-pathogenic bacteria that are derived from the alimentary tract and are able to improve host colonic microenvironment (123). The precise probiotics mechanism of action has yet to be fully clarified. Potential mechanisms on the epithelial barrier include a limited bacterial movement across the mucus layer through increasing mucin expression and secretion by goblet cells, an increased production of AMPs, and an enhanced TJs stability. Overall, these mechanisms reduce epithelial permeability to intraluminal pathogens and toxins (148). Furthermore, probiotics influence mucosal immunity by increasing levels of IgA-producing cells in the lamina propria and promoting secretion of sIgA into the luminal mucus layers avoiding bacteria colonization of the epithelium (148). Through a cascade of signaling events, probiotics enhance the production and secretion of anti-inflammatory cytokines, including IL-10 and transforming growth factor β (TGFβ) by T Regs (117). In small animal models, several reports have suggested that probiotics enhance the local and systemic immune system through an increased activity of IgA, T-cells, macrophages, T helper1 (Th1)-cytokines, as well as the modulation of gut-associated lymphoid tissue, and natural killer cell cytotoxicity (149). Probiotics might also affect the intestinal microbiota and hence limit intestinal bacteria overgrowth and the production of lipopolysaccharides (123).

The beneficial effects of probiotics have been reported in several situations, such as food allergies (150), immune disorders (151), prevention of intestinal tumors (152), prevention of body weight loss in animal models (153), and IBD (154). In fact, probiotics have gained big interest during the last decade as treatments to maintain intestinal homeostasis and reduce specific GI symptoms (155). Although it has been demonstrated that the use of probiotics in humans is relatively safe, some studies have questioned about the administration of a huge quantity of bacteria into a host (156). To avoid this potential risk, instead of using the whole live microorganisms, only probiotic-derived beneficial molecules have been administered. For example, a recombinant 40 kDa soluble protein derived from *Lactobacillus rhamnosus* GG (LGG) was able to reproduce the antiapoptotic effect of the bacterium *in vitro* (157). Importantly, the delivery of LGGp40 to the colon *in vivo* using a pectin/zein hydrogel bead system was able to ameliorate DSS-induced intestinal injury as well as oxazolone-induced Th2-driven colitis (157).

On the other hand, prebiotics are non-digestible food components that contribute to host health by inducing specific changes in the composition and in the activity of intestinal microflora (158). They are fermented oligosaccharides, such as fructooligosaccharides, galactooligosaccharides, lactulose, and inulin, which stimulate the growth of beneficial gut bacteria (159). Due to their composition, they can only be absorbed in the colon, where they ferment into SCFAs and lactate (160), which are crucial energy sources for the host (161). The efficacy of prebiotics in IBD has been studied *in vitro* (162) and in animal models (DSS- and TNBS-induced colitis) (163, 164). However, there are few human studies limited by a small number of patients (165). For example, in a recent study, UC patients were treated with mesalazine and randomly assigned to receive either oligofructose-enriched inulin or placebo. Authors showed that the supplemented groups had lower fecal calprotectin than controls. As fecal calprotectin is an inflammatory marker, they concluded that prebiotics can reduce inflammation in UC patients (166).

Since probiotics are becoming a new therapeutic option, it is necessary to determine which strains have the greatest efficacy, if they are more effective alone or in conjunction with other pro or prebiotics, and what is their half-life in the GI tract. In fact, it is likely that a combined treatment with probiotics and prebiotics, called synbiotic therapy, can have a stronger effect on intestinal diseases than probiotics or prebiotics alone (123). It is also likely that the probiotic, prebiotic, or synbiotic combination will not be suitable for all patients, but the treatment will depend on the individual microbiota composition (123).

In agreement with the effect of the microbiota on the intestinal permeability, a novel treatment option for IBD has gained interest, i.e., fecal microbiota transplantation (FMT) (167). Fecal transplantation (or bacteriotherapy) is the transfer of stool from a healthy donor into the GI tract of a patient. This strategy has been originally designed for the treatment of recurrent *C. difficile* colitis (168). *C. difficile* colitis is a complication of antibiotic therapy that may be associated with diarrhea, abdominal cramping, and sometimes fever (168). FMT appears to be the most effective intervention available for refractory *C. difficile* infection (r*CD*I). In fact, metronidazole, vancomycin, and fidaxomycin (antibiotics used for the treatment of this infection), fail in 30% of treated individuals, causing a relapse of the infection (168). This was clearly demonstrated by an important paper describing a clinical trial that used FMT for r*CD*I (169). A total of 120 patients were enrolled, and they were treated with fecal transplant or vancomycin. The trial was interrupted in advance due to the difference between the two groups. Indeed, fecal transplant was three to four times more effective in eradicating the infection compared to the antibiotic. Fecal infusions cured 13 out of 16 patients (81%), whereas only 7 out of 26 patients (27%) treated with vancomycin obtained the same result (169). Thus, FMT has been shown to be effective in treating relapsing or r*CD*I, but practical barriers and safety concerns have limited its widespread use (170). The majority of reported FMT procedures have been performed with fresh stool suspensions from related donors using delivery by colonoscopy. The use of fresh donations requires prior identification and screening of a suitable donor, thus precluding the use of FMT in acute situations (170). Donor selection is a crucial aspect for FMT. In fact, higher compliance has been reported if FMT is allowed from a donor chosen by the patient. Nonetheless, donations from healthy volunteer donors obtained by an unbiased selection resulted in lower incidence of viral infections and better genetics of the microbiota (171). In fact, it has been reported the case of a woman successfully treated with FMT with stools from a healthy but overweight donor that, later, developed new-onset obesity. The link between obesity and FMT transplant could be the result of multiple factors, including the resolution of *CD*I with subsequent increased appetite and the concurrent treatment of *Helicobacter pylori*. Nonetheless, the same has been observed in animal models that acquired an obese phenotype following the transplant of the microbiota from an obese donor (172). Furthermore, it is likely that the weight gain in the case reported was influenced by a combination of genetic factors derived from the donor and recipient microbiota (173).

It was recently described the successful use of frozen FMT inocula for the treatment of *CD*I. Carefully screened healthy volunteer donors were used to create capsules that prevent the need for invasive procedures for FMT administration, avoid procedure-associated complications, and significantly reduce the cost of the treatment (170). The major limitation of this study is the small sample size, the lack of placebo or active comparator, and the short follow-up that precludes the assessment of long-term immunological effects and onset of latent infections (170). Currently, several aspects limit the scientific value of the described protocol; nonetheless, it is a groundbreaking trial that will hopefully be validated in the near future.

Results from these analyses suggest that FMT is generally tolerable and safe. In fact, symptoms, such as a predominantly self-limiting fever, can be considered a consequence of the administration procedures themselves. Rigorous screening of the donor and donor stool remains particularly important to implement FMT efficacy (167). The approach to FMT in IBD is still in its infancy, and much work remains to be done in order to clarify its ultimate utility. After all, the most efficient FMT delivery strategy and frequency, as well as the definition of an exact mix of prebiotics and probiotics to be transplanted remains to be further investigated (167).

## Concluding Remarks

Increased intestinal permeability can be related to different mechanisms; the opening of pores in the tight junctional complexes, an increased rate of transcytosis of antigens or ICs, the final stage of inflammation, apoptosis, and ulceration. All of these mechanisms cause the entry of luminal antigens into the lamina propria leading to the initiation of inflammatory immune responses that, if protracted, could become a chronic intestinal inflammatory syndrome. Indeed, a common feature of the intestinal diseases is the failure to contain the luminal content. Paracellular or transcellular pathways lead to chronic inflammation if not correctly regulated. Although the majority of dietary proteins are totally degraded by digestive enzymes and are absorbed in the form of nutrients, some can resist both the low pH of the gastric fluid and proteolytic enzyme hydrolysis. This means that large immunogenic peptides or intact proteins are capable of reaching the intestinal lumen by the different pathways described before. Intestinal barrier function is regulated by multiple components. Among these, an emerging role is depicted for nutrition. The role of different dietary antigens in the regulation of intestinal permeability is in part contradictory. While some compounds such as gliadin impair the TJ barrier ultimately leading to CD, others such as Gln and polyphenols enhance and protect TJ barrier integrity. The latter category of food could be used as therapeutic tools for diseases associated with barrier defects. The effects of dietary compounds as preventive or therapeutic agents in different intestinal diseases have to be studied taking into account the complexity of the intestinal environment. The integrity of the epithelial cell monolayer is one of the players involved, and, at the moment, we still largely ignore whether barrier defects are the trigger or the effect of chronic inflammatory syndromes. A further level of complexity is determined by the variety of modifications that change the chemical structure of the nutritionally derived compounds along the way through the intestine.

Composition of dietary intake can have significant impact on the microbiota, and consequently on the epithelial barrier. Considering bacteria fermentation, diets enriched in milk fat can promote the increase in barrier permeability and bacterial translocation along with the decrease in TJ proteins and TER. On the contrary, diets, including probiotic bacterial species or prebiotic fibers, strengthen the epithelial barrier by increasing TJ proteins and TER and decreasing permeability and bacterial translocation, thus, preventing or ameliorating the inflammatory state. It remains to be determined by the link between nutrition and microbiota composition. This is a challenging task as the influence of factors, such as genetics, hygiene, and living conditions, are not taken into account in studies comparing distinct populations.

Understanding the intricate relationship between epithelial barrier, microbiota, and diet will contribute to design new preventive/therapeutic approaches for GI diseases, particularly IBD. This is becoming in part true if we consider the use of fecal therapy instead of classical antibiotics treatment for r*CD*I. Nonetheless, this new therapeutic approach needs to be further investigated, in particular, for the definition of precise parameters for the donor choice.

Deep analysis of the microbiota is now possible due to faster sequencing techniques and improved bioinformatics tools even if the major challenge remains the ability to discriminate between healthy and disease microbiota state. In fact, substantial inter- and intra-individual variations in addition to age-related changes in the composition of the intestinal microbiota were identified providing an additional level of complexity to this intricate pattern.

In the recent years, the challenge for the human race was to obtain enough food to provide sufficient nutrients to the body. Nowadays, in vast regions of the planet, the challenge is to sustain the body with the right combination of nutrients able to prevent chronic inflammatory syndromes. We knew the importance of the intestinal permeability, we then realized the importance of the cross talk between the intestinal epithelium and the immune system, and we are just starting to realize the pivotal role of the microbiota for human health. A further level of complexity will need to be addressed in the near future; in particular, we will need to understand the cascade of events that lead to chronic inflammatory syndromes. *In vitro* studies are still required to dissect the effects of single nutritional components as well as their effects when combined. Animal models will be required to better understand the effects of nutritional strategies for the prevention/treatment of chronic inflammatory syndromes. We still need to shed light on the axis between microbial communities and the nutritional intake as well as on the axis between microbial communities and post-modifications of the nutritional compounds. These modifications may severely impact the biological effects of the nutrients. Finally, in light of what was discovered by Fonseca and others (174), trials based on food components as complementary approach, should consider a working hypothesis made of three consecutive steps. The first should be an antibiotic approach to eliminate the pre-existing microbiota, the second should be the administration of a selected mix of prebiotics and probiotics to create a known microbiota, and then, the final step should be the nutritional intervention.

The switch from acute to chronic disease characterized the last century. Health systems are still adjusting to the need for long term and personalized medicine that will consider nutrition much more than just food intake.

## Author Contributions

All authors contributed to the manuscript by constructive ­discussions and proof reading.

## Conflict of Interest Statement

The authors declare that the research was conducted in the absence of any commercial or financial relationships that could be construed as a potential conflict of interest.
